# Editorial: Stance-taking in embodied and virtual interaction

**DOI:** 10.3389/fpsyg.2025.1636418

**Published:** 2025-07-15

**Authors:** Elisabeth Zima, Geert Brône, Kurt Feyaerts, Silva Ladewig

**Affiliations:** ^1^Department of German Studies, University of Freiburg, Freiburg, Germany; ^2^Department of Linguistics, University of Leuven, Leuven, Belgium; ^3^Sign Lab, Georg-August-Universitat Gottingen, Gottingen, Germany

**Keywords:** stance-taking, multimodality, embodied interaction, virtual interaction, gesture, multimodal packages, cognitive underpinnings

A plethora of conversation-analytic, discourse-functional, cognitive and psycholinguistic studies from recent years emphasize the central role of stance-taking in social interaction: “Whenever we engage in interaction, we are taking stances: there is never a time out from the social action of taking stances and adopting positions” (Du Bois and Kärkkäinen, 2012, p. 438). This act of positioning can involve three different axes, which are often considered to represent different types of stance-taking: epistemic, affective, and deontic. While epistemic stance-taking is understood as “the stance that a participant in an interaction takes with their turn in relation to a certain object of knowledge” (Deppermann, 2018, p. 121, our translation), e.g. by expressing (un)certainty, deontic stances refer to judgements about the necessity or desirability of actions, events, suggestions or similar *stance objects* (Du Bois, 2007), e.g. in cases of invitations or orders. Finally, the category of affective stances includes attitudinal and evaluative expressions which, according to Ochs (1996, p. 104), express “mood, attitude, feeling, and disposition, as well as degrees of emotional intensity vis-à-vis some focus of concern”.

Following Du Bois (2007) and Jaffe (2009), stance-taking does not merely reside in the expression of one's own feelings and opinions but crucially serves to position oneself and others and thus to negotiate the interpersonal relationship by either expressing (dis-)alignment with other participants and their stances. While aligning stances serve the purpose of creating or emphasizing commonalities and thus enforcing and displaying togetherness (*interpersonal alignment*, Du Bois, 2007), disalignment may serve the purpose of setting oneself apart from others. This is reminiscent of Stivers' concept of affiliation, which she defines as the actions by which “the hearer displays support of and endorses the teller's [or interactant's, our addition] conveyed stance” (Stivers, 2008, p. 35). In contrast, disaffiliative stances express an attitude that diverges from the stance expressed by interaction partners (also Steensig and Drew, 2008; Drew and Walker, 2009 and especially Stivers, 2010, 2020). As social actions, however, affiliation and disaffiliation are not to be understood as dichotomous categories, but as two end points of a continuum (cf. Thompson et al., 2015, p. 176–178 and their analysis of stronger and weaker affiliative and disaffiliative evaluations). Similarly, the triadic relationship between stance(-taking) subjects and objects (cf. “the stance triangle”, Du Bois, 2007) is not a static construct, but fundamentally dynamic in nature and subject to interactional negotiation.

Research paradigms such as Conversation Analysis, Interactional Linguistics and psycholinguistics have a long track record in the study of stance-taking as a socially contextualized and recognized interpersonal phenomenon, focusing on the lexical and grammatical resources that language users have at their disposal to communicate stance. Among these linguistic resources, special attention has gone to *pre-positioned elements (*Auer and Lindström, 2016) such as *well, okay, I don't know* etc. (Auer and Uhmann, 1982; Pomerantz, 1984; Szczepek Reed, 2015; Pekarek Doehler et al., 2021), but also to concessive constructions, modal verb constructions, and hedges. These verbal means to express stance have been studied extensively in different communicative settings (from spontaneous face-to-face communication to institutional and mediated forms of interaction), from different disciplinary angles (Interactional Linguistics, Ethnomethodology, Cognitive Psychology, HCI Research, etc.) and using different empirical methods (from controlled experiments to qualitative and quantitative corpus analysis).

However, the expression of stance - both in *face-to-face* and in virtual communication - is by no means limited to verbal means. Recent studies have explored the ways in which embodied resources such as hand gestures (see also the contributions to this Topic by Cienki, Inbar, Ladewig), body movements (Betz and Gubina), body posture, facial expressions and eye gaze (de Vries et al.; and Laner, and Clift in this Topic) play a role in the expression of stance (see Andries et al., 2023 for a systematic literature review). The goal of this Research Topic is to highlight this recent trend in the study of stance-taking and to present a state-of-the-art collection of original research that zooms in on either the co-occurrence of (i.e., “multimodal packages”, Mondada, 2014) or interdependence between different semiotic resources (i.e. the sequential relationship within or across speakers).

The contributions to this Topic take a broad perspective on multimodal interaction. They unite a variety of methodological approaches, ranging from the study of naturally occurring interactions to carefully controlled experiments that tap into the cognitive underpinnings of multimodal stance-taking. Apart from the basic question on how stance is multimodally construed and negotiated in spoken and signed face-to-face interaction, the Research Topic also explores the strategies that interlocutors employ to express stance in mediated forms of interaction. It presents original research, with a particular focus on empirical studies that adopt a decidedly multimodal approach to this phenomenon.

Except for the contributions by Cienki, who focuses on stance-taking in gestures by simultaneous interpreters, and Dancygier and Vandelanotte, who study memes in online discourse, all articles in this Topic are concerned with the interactional negotiation of stance and its sequential embeddedness in different interactional contexts. These range from various forms of face-to-face interactions, including interactions in the less-studied side-by-side formations (Pfänder and Pfänder, Laner), to educational settings (Li), neurological consultations (Logren et al.), political debates (Tabacaru), and parliamentary discourse (Müller et al.) They cover the whole spectrum from affiliative to disaffiliative stance-taking (Stivers, 2008), focusing on e.g. strategies to enhance intersubjectivity (Li), humor and mockery (Tabacaru, de Vries et al.), the management of conflict (Clift) and divergent stances (Logren et al., Pfänder and Pfänder).

More specifically, the contributions by Inbar, Ladewig, Pfänder and Pfänder, Tabacaru and de Vries et al. explore how resources from different semiotic channels are combined to form recurrent multimodal packages (Mondada, 2014). Dancygier and Vandelanotte, Ladewig, Müller et al., Pfänder and Pfänder, Rühlemann and Trujillo focus on the interplay between the local, situational dimensions of stance-taking and its effects in terms of the negotiation of interpersonal relationships, emotion work and identity construction. The studies by Dancygier and Vandelanotte, Ladewig, and Li are furthermore concerned with the impact of medium—or in Laner's case the spatial formation and the interaction with the surrounding nature—on the strategies that interlocutors employ to express stance. Finally, the studies by Cienki, Rühlemann and Trujillo as well as Zheng and Sun broaden our perspective on the interactional phenomenon of stance-taking by inquiring into the cognitive processes underpinning stance-taking in interaction with Cienki also focusing on cross-linguistic differences.

In sum, this Research Topic is the first collection of original work that is specifically devoted to the study of how different semiotic resources contribute to stance-taking activities in a wide array of (interactional) contexts. What the 15 contributions to this Research Topic show, is that the topic of multimodal stance-taking can and must be approached from various angles, each of which adds to a better understanding of what is one of the most fundamental social activities realized in/through language and embodied behavior. While qualitative studies in the tradition of Conversation Analysis and Interactional Linguistics provide us with relevant insights into the sequential organization and social dynamics of multimodal stance-taking, the more quantitatively oriented experimental and corpus-based studies help us in identifying patterns and relevant factors that drive the expression and negotiation of stance. It was the explicit intention of this Research Topic to unite these different perspectives and thus to set the agenda for future research on the multifaceted nature of stance-taking in embodied and virtual interaction.
